# High PD-L1 expression was associated with poor prognosis in 870 Chinese patients with breast cancer

**DOI:** 10.18632/oncotarget.5583

**Published:** 2015-09-10

**Authors:** Tao Qin, Yin-duo Zeng, Ge Qin, Fei Xu, Jia-bin Lu, Wen-feng Fang, Cong Xue, Jian-hua Zhan, Xin-ke Zhang, Qiu-fan Zheng, Rou-jun Peng, Zhong-yu Yuan, Li Zhang, Shu-sen Wang

**Affiliations:** ^1^ Sun Yat-sen University Cancer Center, The State Key Laboratory of Oncology in South China, Collaborative Innovation Center for Cancer Medicine, Guangzhou, Guangdong, P. R. China; ^2^ Sun Yat-sen University Sun Yat-sen Memorial Hospital, Guangzhou, Guangdong, P. R. China

**Keywords:** PD-L1, breast cancer, ER, PR, prognosis, nomogram

## Abstract

**Background:**

To investigate the role of PD-L1 expression in tumor recurrence and metastasis in Chinese patients with breast cancer.

**Methods:**

Suitable tissue samples were available from 870 patients with breast cancer. Paraffin-embedded tumor sections were stained with PD-L1 antibody. The correlations between PD-L1 expression and clinical characteristics, ER/PR/HER2 status and survival parameters were analyzed. Kaplan-Meier and univariate Cox proportional hazards model analyses were used to compare the survival of patients with high PD-L1 expression and patients with no PD-L1 expression.

**Results:**

The median follow-up time was 98 months(range, 17–265 months). The positive rate of PD-L1 expression in breast cancer was 21.7% (189/870). PD-L1 high expression was inversely associated with larger tumor size, higher tumor grade, more positive lymph node number, as well as negative ER and PR status. PD-L1 expression was particularly higher in TNBC compared with non-TNBC, although no statistical significance was observed. Nomogram logistic regression results based on clinical and pathological features showed that the following factors were more likely associated with high PD-L1 expression: patient age younger than 35 years, larger tumor size, lymphovascular invasion and advanced stage. Our data indicated that patients with high PD-L1 expression had poor DFS, DMFS and overall survival compared with those with no PD-L1 expression. Univariate Cox proportional hazards model analysis showed that PD-L1 was an independent prognostic factor for tumor prognosis.

**Conclusions:**

PD-L1 expression is an important indicator of unfavorable prognosis in breast cancer patients.

## INTRODUCTION

Breast cancer is currently the second leading cause of tumor-related death for females worldwide [1]. Despite the development of treatments for breast cancer, more than 50% of invasive breast cancer patients have developed distant metastases within ten years, causing treatment failure [2]. Interestingly, in recent years, immune therapy has become an emerging effective treatment for several cancers.

Recently, extended adjuvant endocrine therapy was shown to benefit patients with hormone receptor-positive breast cancer in the large sample size randomized ATLAS study [3]. However, the recurrence of breast cancer remained high in realistic clinical practice. Therefore, finding an effective biomarker to select patients with breast cancer who are at a high risk for tumor recurrence or metastasis is an urgent task.

The risk of tumor recurrence is reasonable within the 5-yearfollow-up period following treatment with adjuvant tamoxifen; however, a number of patients with luminal B/HER2 negative subtype tumors presented tumor recurrence after the 5-year period following treatment with tamoxifen [2]. Several predicting tools including Breast Cancer Index (BCI), Oncotype DX recurrence score, IHC4 [4], andHOXB13/IL17BR (H/I) [5]were used to predict the risk of late disease recurrence. Although several predicting markers have been developed, no precise factor existed that could predict the long-term survival of breast cancer patients. The primary cause of recurrence or metastases from operable breast cancer was immune resistance produced by the tumor. Immune activity plays a role in cancer metastatic immune checkpoint agonist drugs have been approved for use in treating melanoma. Previously, the PD1/PD-L1 pathway was reported as the primary factor promoting tumor recurrence or metastasis. PD-L1, which is major ligand of PD-1, is expressed in a variety of cancers. Studies have shown PD-L1 inhibition is effective treating in many malignant tumors such as non-small cell lung cancer (NSCLC) [6], renal cancer [7], triple-negative breast cancer [8] and bladder cancer [9, 10].

Tumors can cause changes in the microenvironment and may evoke an imbalance of immunomodulation between tumor growth and host surveillance, finally promoting tumor metastasis. PD-1/PD-L1 checkpoint antibodies help to reconstruct the balance between the host and tumor, resulting in dynamic and durable tumor regression. Nivolumab and pembrolizumab are two anti-PD-1 antibodies that are currently approved for use in clinical treatments for melanoma. MPDL3280A, MEDI4736, and BMS-936559 were investigated for potential treatment of other tumor types such as metastatic melanoma, NSCLC, and breast cancer [11]. Currently, the PD1/PD-L1 pathway in breast cancer has been well studied [12, 13]. However, the molecular regulatory mechanism of PD-L1 in different subtypes of breast cancer remains unknown [12]. As previous studies have indicated, the positive rate of intra tumor PD-L1 expression ranged from 20% to 60% of breast cancer patients [14, 15]. A large randomized study showed that PD-L1 blockade prolonged survival in triple-negative breast cancer that lacked an effective treatment [16]. However, PD-L1 expression reflected inconsistent survival outcomes in breast cancer. Two studies showed that tumors with a high level of PD-L1mRNA expression correlated with significantly better recurrence-free survival in breast cancer patients [17, 18], whereas other studies showed that high PD-L1 expression was significantly associated with poorer survival [14]. Recently, a genomic analysis of the PD-L1 gene in breast cancer showed that the PD-L1 gene was inversely associated with the ESR1 gene in 5,454 breast cancers profiled using DNA microarrays [18]. IHC studies strongly suggested that PD-L1 expression was an unfavorable factor that was associated with decreased disease-free survival and overall survival [14, 15]. In addition, several studies showed that PD-L1 was more highly expressed in triple-negative breast cancer and HER2-positive breast cancer [19]. Therefore, studying PD-L1 expression in Eastern Asian patients with breast cancer is of significance. This study aimed to explore the role of PD-L1 expression in the prognosis of870 Eastern Asian breast cancer patients.

## RESULTS

### Patient characteristics

In total, 870 Eastern Asian patients with invasive breast cancer were enrolled in this study. The median age at diagnosis was 47.0 years (range, 21–84 years). The baseline characteristics of these patients are listed in Table 1. PD-L1 expression was more common in patients with tumors that were larger than 2cm, with lymphvascular invasion, a higher tumor grade, as well as negative ER and PR status.

**Table 1:** PD-L1 expression levels of 870 breast cancer patients

*Variable*	*PD-L1 expression [n(%)]*		*P-value^a^*
	*negative*	*Positive*	
Medianage (years)	47 (21-84)		
Tumor size(mm)			**0.002**
≤20	238 (84.4)	44 (15.6)	
>20	38 (66.7)	19 (33.3)	
Stage			0.257
I	129 (83.2)	26 (16.8)	
II	461 (77.2)	136 (22.8)	
III	91 (77.1)	27 (22.9)	
Histologicalgrade^a^			**0.013**
1	32 (86.5)	5 (13.5)	
2	279 (82.5)	59 (17.5)	
3	370 (74.7)	125 (25.3)	
Positive lymph nodes			0.533
0	334 (78.2)	93 (21.8)	
1–3	190(80.9)	45 (19.1)	
4–9	93 (76.9)	28 (23.1)	
≥10	64 (73.6)	23 (26.4)	
Lymph node ratio			0.470
<0.20	169 (80.1)	42 (19.9)	
0.21≤xx<0.65	120 (78.4)	33 (21.6)	
>0.65	58 (73.4)	21 (26.6)	
Lymphovascular invasion			**0.015**
No	664 (78.8)	179(21.2)	
Yes	16 (59.3)	11 (40.7)	
Receptor status			
Estrogen			**<0.001**
Positive	570 (90.5)	60 (9.5)	
Negative	111 (46.30)	129 (53.8)	
Progesterone			**<0.001**
Positive	555 (90.0)	62 (10.0)	
Negative	126 (49.8)	127 (50.2)	
her2			0.529
Negative	668 (78.4)	184 (21.6)	
Positive	13 (72.2)	5 (27.8)	
Ki67 index^b^			0.028
≤14%	86 (69.9)	37 (30.1)	
>14%	349 (79.3)	91 (20.7)	
Neo-adjuvant chemotherapy			0.428
No	625 (79.6)	170 (21.4)	
Yes	56 (74.7)	19 (25.3)	
Chemotherapy			0.109
No	78 (84.8)	14 (15.2)	
Yes	603 (77.5)	175 (22.5)	
Radiotherapy			0.797
Yes	521 (77.8)	14 (22.2)	
No	23 (74.2)	8 (25.8)	
Endocrinetherapy			**<0.001**
No	192 (61.9)	118 (38.1)	
Yes	489 (87.3)	71(12.7)	
Subtypes			**<0.001**
Luminal A	284 (88.5)	37 (11.5)	
Luminal B/HER2 negative	287 (91.4)	27 (8.6)	
Luminal B/HER2 positive	12 (92.3)	1 (7.7)	
Triple-negative	98 (44.1)	124 (55.9)	

a her2, human epidermal growth factor receptor 2;

b 563 patients with known ki67expression.

### PD-L1 expression and patient baseline clinical characteristics

**Figure 1:**
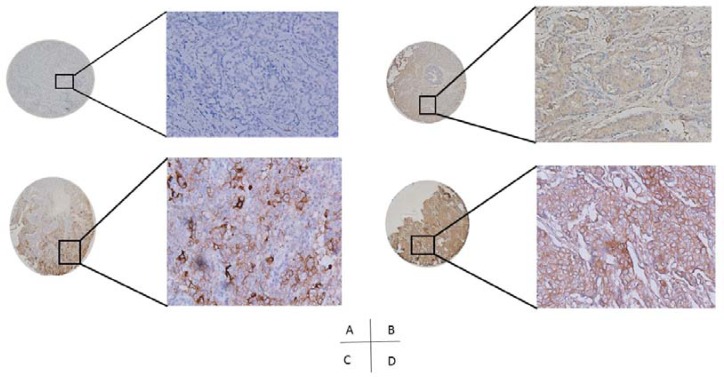
PD-L1 expression in breast cancer tissues (A, PD-L1 negative; B, C&D, PD-L1 positive)

PD-L1 was found at the membrane or in the cytoplasm (or both) of tumor cells by immunohistochemical staining (Figure 1). IntratumorPD-L1 expression was observed in 189 (21.7%) patients. The relation of PD-L1 expression with various clinicopathological parameters is shown in Table 1.

**Figure 2:**
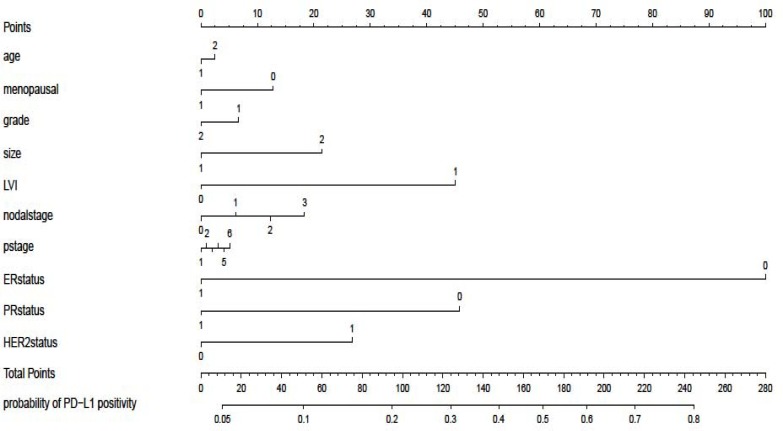
Nomogram predicting patients with PD-L1-positive tumors according to varied clinical characteristics LVI, lymphovascular invasion.

In addition, patient characteristics were more associated with the probability of positive PD-L1 expression. Therefore, we used a nomogram to predict which subtype of patients was more likely to present high PD-L1 expression. Nomogram analysis focused on clinical characteristics to predict PD-L1 positive. The results was to distinguish which subtype of patients with breast cancer should be detect PD-L1 concurrent with ER/PR/HER2 after breast surgery. in addition, our data also showed that patients with the following characteristics were more likely associated with high PD-L1 expression: larger tumor size, lymphovascular invasion, advanced nodal stage, negative ER status, negative PR status as well as HER2 status (Figure 2).

### Relation between PD-L1 expression and prognosis

**Figure 3:**
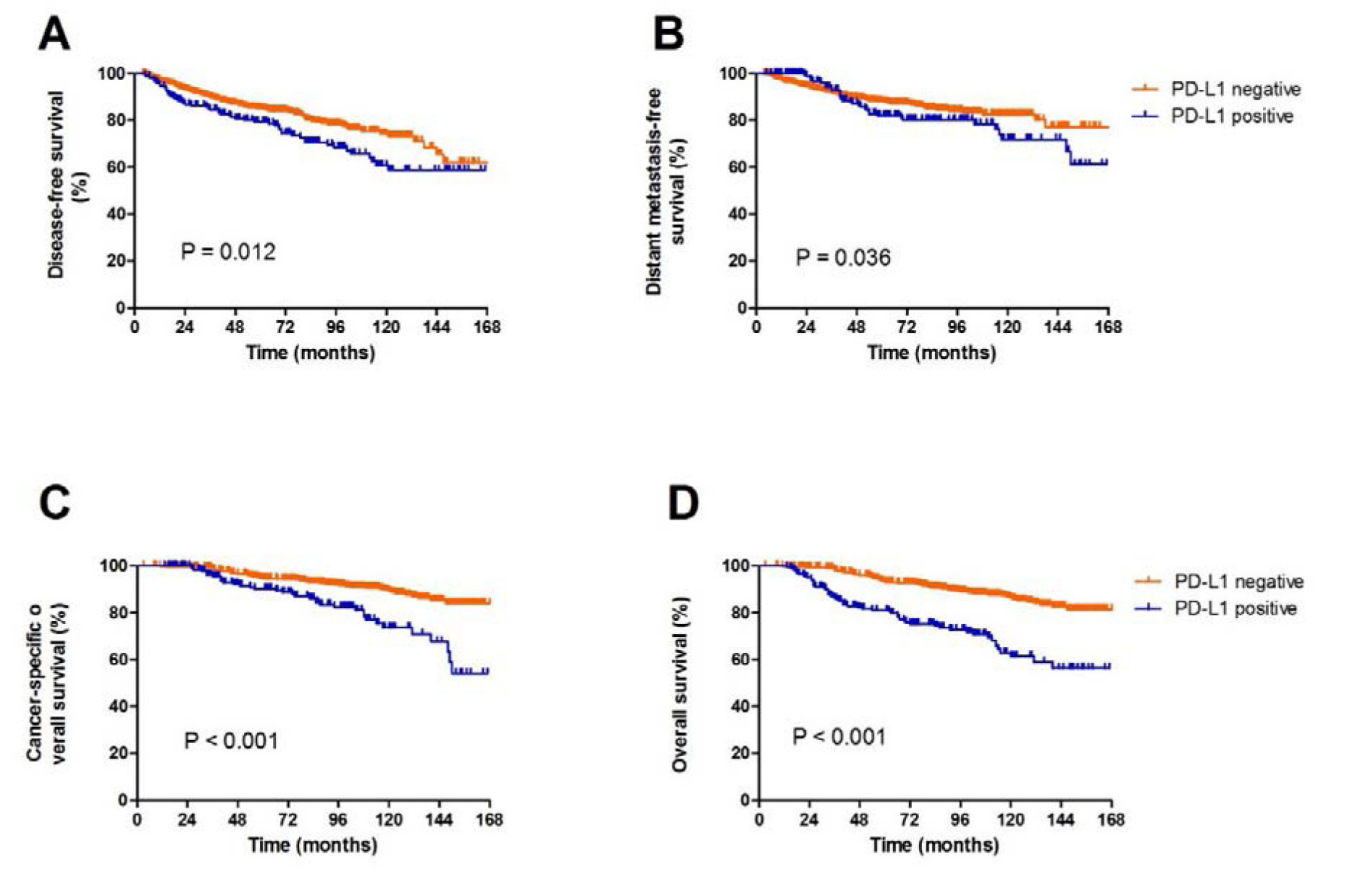
Survival analysis according PD-L1 expression(A, DFS; B, DMFS; C, cancer-specific OS; D, OS)

The median follow-up time was 98 months(range, 17-265 months). PD-L1-positive breast cancer patients had significantly shorter DMFS, DFS and OS values than those of PD-L1-negative patients (Figure 3). The 5-year DMFS for PD-L1-positive patients was significantly poorer than those withPD-L1-negative patients(83% *vs*. 88%, *P* = 0.036). When the patients were stratified in terms of PD-L1 status, the five-year DFS values for PD-L1-positive and PD-L1-negative patients were 78.6% *vs*. 84.9% (*P* = 0.012), respectively. The cancer-specific overall survival for PD-L1 positive patients was significantly poorer than that of PD-L1-negative patients (88% *vs*. 91.5%, *P* < 0.001).

### Univariate and multivariate analyses

PD-L1 expression and patient's characteristics including age, tumor size, tumor grade, positive lymph node number, lymph node ratio, ER status, PR status, HER2status were included to perform univariate and multivariate analyses.

Statistically significant predictors of DFS within the univariate analysis are listed in Table 2. In univariate survival analyses, larger tumor size, positive lymph-node status, PD-L1 expression revealed unfavorable DFS for breast cancer patients.

**Table 2:** Univariate and multivariate analyses for DFS

*Variables*	Univariate				Multivariate			
	*HR*	95% *CI*		*P-value*	*HR*	*95% CI*		*P-value*
		*Lower*	*Upper*			*Lower*	*Upper*	
Age (continue)	0.991	0.977	1.005	0.203				
Tumor size(>20 vs. ≤20 mm)	2.530	1.688	3.791	**<0.001**	1.960	1.302	2.951	**0.001**
Grade(III vs. I-II)	1.132	0.861	1.488	0.373				
LN status(pos vs. neg)	4.084	2.857	5.839	**<0.001**	3,.731	2.604	5.346	**<0.001**
ERstatus (pos vs. neg)	0.900	0.652	1.242	0.521				
PR status (posvs. neg)	0.943	0.685	1.299	0.721				
HER2 status (pos vs. neg)	1.181	0.971	1.437	0.097				
ki67 (>14% vs. ≤14%)	1.283	0.775	2.214	0.333				
PD-L1status (pos vs. neg)	1.503	1.091	2.071	**0.013**	1.386	1.003	1.916	**0.048**
TNBC (yes vs. no)	0.987	0.704	1.384	0.942				

Abbreviations: LN, lymph node; pos, positive; neg, negative; HER2, human epidermal growth factor receptor type 2; TNBC, triple-negative breast cancer.

In addition, a larger tumor size, positive lymph-node status, and PR-negative, triple negative and PD-L1-positive expression were associated with poorer OS (Table 3). In the multivariate analysis, a tumor size larger than 2 cm, positive lymph-node status and PD-L1-positiveexpression proved to be independent negative prognostic factors for both DFS and OS (Table 3).

**Table 3:** Univariate and multivariate analyses for OS

*Variables*	Univariate				Multivariate			
	*HR*	95%*CI*		*P-value*	*HR*	*95% CI*		*P-value*
		*Lower*	*Upper*			*Lower*	*Upper*	
Age (continue)	1.005	0.990	1.021	0.477	1.249	0.685	2.276	0.469
Tumor size(>20 vs. ≤20 mm)	2.648	1.679	4.174	**<0.001**	1.880	1.183	2.990	**0.008**
Grade(III vs. I-II)	1.242	0.916	1.683	0.163				
LN status(pos vs. neg)	4.718	3.080	7.226	**<0.001**	2.222	1.9056	2.592	**<0.001**
ER (pos vs. neg)	0.871	0.755	1.005	0.059				
PR(posvs. neg)	0.797	0.687	0.926	**0.003**	0.908	0.420	1.963	0.806
HER2 status (pos vs. neg)	1.118	0.881	1.419	0.360				
ki67 (>14% vs. ≤14%)	0.831	0.487	1.417	0.497				
PD-L1 (pos vs. neg)	2.262	1.598	3.203	**<0.001**	1.788	1.195	2.674	**0.005**
TNBC (yes vs. no)	1.454	1.003	2.108	**0.048**	1.398	0.606	3.225	0.432

Abbreviation: LN, lymph node; pos, positive, neg, negative; TNBC, triple-negative breast cancer.

## DISCUSSION

We investigated the prevalence and significance of PD-L1 expression in breast cancer. In this study of 870 breast cancer patients, the total positivity of PD-L1 was greater than20%. PD-L1 high expression was inversely associated with large tumor size, higher tumor grade, more positive lymph node number, higher lymph node ratio, negative ER/PR status. PD-L1 expression was particularly higher in TNBC compared with non-TNBC. As our data demonstrated, patients with positive PD-L1 expression had significantly decreased survival compared to those with no PD-L1 expression. Cox proportional hazards model analysis indicated that PD-L1 expression was a strong independent prognostic factor for patient prognosis.

Programmed death 1 (PD-1) is a co-inhibitory receptor that is expressed on the membranes of activated T and B cells [6] and that plays an important role in tumor immune escape [7, 8]. The major ligand for PD-1 is PD-L1, which is expressed in a variety of cancers [9]. Adaptive immune responses that includePD1/PD-L1 expression are associated with breast cancer relapse. PD1/PD-L1 is an important axis that plays important roles in the infiltration of various immune effectors and in the propensity to relapse with metastatic disease. Recent evidence suggests that activation of the PD-1/PD-L1 pathway represents one mechanism that allows tumors to elude the host immune system [16-18]. Previous studies have reported that PD-L1 is involved in the negative regulation of immune response binding to PD-1 receptor and results in cancer cells evading the host immune surveillance, finally promoting metastasis [10-12].

PD-L1 expression was evaluated as a predictor of unfavorable prognosis for many other malignant cancers such as NSCLC, melanoma, renal cancer, glioblastoma (GBM), ovarian cancer, and colon cancer. Regarding breast cancer, the reported positivity of PD-L1 expression in tumor cells varied. A recent study showed that PD-L1 expression by immune cells was observed in 6% of tumors, while PD-L1 expression by tumor cells occurred in only 1.7% of a total of 3796 breast cancer patients [20]. However, in our study, we showed that PD-L1 expression in breast tumor cells occurred in 21.7% of all patients. Our results were consistent with PD-L1 expression results of previous reports. Moreover, statistical analysis found that PD-L1 was associated with many tumor characteristics of breast cancer. In addition, clinical characteristics closely correlated with PD-L1 expression. Ghebeh analyzed 44 patients and found that PD-L1 expression was significantly higher in ER-negative tumors, PR-negative tumors and higher-grade tumors [15]. However, due to the sensitivity ofdetectionforPD-L1, the rate of PD-L1 expression differed. Another study using immunohistochemical methods showed that high PD-L1 mRNA expression levels were more common in patients with the following characteristics: a larger tumor size, high proliferation, high tumor grade, and ER-negative and PR-negative status [18]. Our data were consistent with previous studies and demonstrated that positive PD-L1 expression was associated with tumor grade and with ER and PR status. Moreover, patients with tumor size and LVI had a higher proportion of positive PD-L1 expression. The results of our study were consistent with previous reports.

PD-L1 expression varied in different subtypes of breast cancer. One study showed higher positive rates of PD-L1 expression in different types of tumor cells,20% in HER2-positive cells, 33% in luminal subtype cells and up to 59% in triple-negative breast cancer cells [19]. PD-L1 mRNA expression levels were higher in HER2-positiveand in basal and HER2-enriched subtypes than in other subtypes [18]. PD-L1 expression was the highest in TNBC, in contrast to a recent study that reported the highest frequency in HER2-positive breast cancers [12, 14]. In our study, patients with TNBC seemed to have a higher proportion of positive PD-L1 expression compared with patients with non-TNBC breast cancer; however, this result was not statistically significant. Our results showed that the percentages of PD-L1 expression in luminal A, luminal B/HER2 negative, luminal B/HER2 positive and TNBC were 11.5%, 8.6%, 7.7% and 55.9%, respectively. Our data indicated that patients with TNBC had a similar rate of PD-L1 expression compared with previous reports.

In addition, several studies found that other malignant oncogenic genes regulated the expression of PD-L1; for example, the EGFR pathway induces PD-L1 expression [21-23]. One limitation of the present study is that no relation was found between the breast cancer-associated gene HER2 and PD-L1. Previous studies have shown that PD-L1protein or mRNA expression levels in breast cancer tumor samples were associated with large tumor size, high tumor grade, more positive lymph-node involvement, ER-negative status, PR-negative status, ERBB2-positive status, and high proliferation, as well as unfavorable molecular subtypes such as HER2-enriched breast cancer or TNBC [12, 14, 15, 17, 24]. However, a recent study showed that high PD-L1 mRNA levels were associated with better prognosis [18]. An *in vitro* study indicated that PD-L1 expression was shown to be higher in a basal type of breast cancer cells than in luminal type cells [13]. In this study, our analysis of breast tumor samples from 870 patients, we demonstrated that PD-L1 was more common in patients with the following clinical characteristics: larger tumor size, more positive lymph node involvement, higher historically tumor grade, higher ki67 index, more LVI and negative relation with both ER and PR.

Apart from our analysis, no studies have used a model to predict the probability of PD-L1 expression according to clinical variables. In our study, we used a logistic regression model help to determine those patients who were likely to have high PD-L1 expression. This finding has not reported previously. This model will help to select those specific patients that should be tested for PD-L1 expression and may be applicable for use in clinical practice.

In our study, which compared between patients with PD-L1positive and patients with PD-L1 negative, patients with positive PD-L1 expression had significantly poorer clinical outcomes including DFS, DMFS, OS and cancer-specific OS. Patients with positive PD-L1 expression had almost two times higher risks of tumor recurrence, metastasis and cancer-related death. Several pathways may activate thePD-L1 pathway.

Notably, we concluded that high PD-L1 expression is inversely associated with large tumor size, tumor grade, lymph node positive number, and ER and PR status. Furthermore, PD-L1 expression is an important prognostic indicator of unfavorable prognosis in breast cancer patients. Finally, a nomogram model is useful for predicting high PD-L1expression levels.

## MATERIALS AND METHODS

### Ethical statement

This study was approved by the Sun Yat-sen University Cancer Center review board.

### Patients

All patients were diagnosed with invasive ductal breast cancer with pathological confirmation at our institution from April 2000 to April 2012. All patients underwent breast conservation therapy or mastectomy. These patients received adjuvant chemotherapy and/or radiotherapy as needed according to the routine clinical practice of our center. Patients with ER/PR-positive tumors received adjuvant endocrine therapy.

### Tissue samples

All slides were cut from a pre-existing invasive ductal carcinoma maintained by the Moffitt tissue core facility (breast 2B). Patient clinical information including age, tumor size, lymph node involvement, tumor grade, and ER, PR, and HER2 status was collected. Additional data including neo-adjuvant chemotherapy, chemotherapy, radiotherapy and endocrine therapy were also collected.

### Immunohistochemical staining

Slides were stained using a Ventana Discovery XT automated system (Ventana Medical Systems, Tucson, AZ) with proprietary reagents according to the manufacturer's protocol. Briefly, slides were deparaffinized on the automated system with EZ Prep solution (Ventana). A heat-induced antigen retrieval method was used withCell Conditioning 1 solution (Ventana). The concentration of rabbit primary antibody that reacts to PD-L1 (Cell Signaling Technology, Beverly, MA) was 1:100 in Dako antibody diluent; slides were incubated with this antibody overnight at 4°C. Then, the slides were incubated with Ventana Omni Mapanti-rabbit secondary antibody for 60 min. AVentana Chromo MapKit was used for antibody detection, and then the slides were counterstained with hematoxylin. Next, the slides were dehydrated and cover slipped as per normal laboratory protocol. All slides were independently examined by two pathologists; both of whom had no prior knowledge of the clinical parameters of the patient. Discrepancies were resolved through the simultaneous re-examination of the slides using a double-headed microscope by both pathologists. PD-L1-positive scoring denoted staining of over 5% of the tumor cell membrane with or without cytoplasm staining.

### Statistical analysis

The patient distribution and clinical features between PD-L1-positive and PD-L1-negative tumors were compared by chi-square test, Wilcoxon rank-sum test, or two-sample t-test as appropriate. The primary endpoint for this analysis was disease-free survival (DFS), which is defined as the length of time from the date of surgery on the primary tumor to local, regional, or distant recurrence or to death from any cause. The second endpoints were overall survival (OS) and distant metastasis-free survival (DMFS). OS is defined as the length of time from the date of surgery on the primary tumor to death from any cause, or to time of last visit. DMFS is defined as the length of time from the date of surgery on the primary tumor to time of distant disease recurrence. Survival curves based on PD-L1 expression were estimated using the Kaplan–Meier product-limit method and compared by log-rank test. Univariate Cox proportional hazards models were fit to identify factors significantly related to DFS and OS. To assess whether the expression of PD-L1 by tumor cells was an independent predictor of survival, a multivariate Cox model was constructed to adjust for other patient/clinical characteristics that were significant in the univariate analyses. Two-way interaction terms between PD-L1 expression and other factors in the multivariate Cox model were also assessed. A nomogram was used to predict the positive probability of PD-L1. All analyses were two-sided, and significance was set at a p-value of 0.05. Statistical analyses were performed using SPSS statistics software version 19 and R software 2.15.3.
